# New resources for functional analysis of omics data for the genus *Aspergillus*

**DOI:** 10.1186/1471-2164-12-486

**Published:** 2011-10-05

**Authors:** Benjamin M Nitsche, Jonathan Crabtree, Gustavo C Cerqueira, Vera Meyer, Arthur FJ Ram, Jennifer R Wortman

**Affiliations:** 1 Institute of Biology Leiden, Leiden University, Sylviusweg 72, 2333 BE Leiden, The Netherlands; 2 Institute for Genome Sciences, University of Maryland School of Medicine, Baltimore, MD 20850, USA; 3 Microbial Informatics, Broad Institute, 320 Charles Street, MA 02141, USA; 4 Kluyver Centre for Genomics of Industrial Fermentation, PO Box 5057, 2600 GA, Delft, The Netherlands; 5 Institute of Biotechnology, Berlin University of Technology, Gustav-Meyer-Allee 25, 13355 Berlin, Germany

## Abstract

**Background:**

Detailed and comprehensive genome annotation can be considered a prerequisite for effective analysis and interpretation of omics data. As such, Gene Ontology (GO) annotation has become a well accepted framework for functional annotation. The genus *Aspergillus *comprises fungal species that are important model organisms, plant and human pathogens as well as industrial workhorses. However, GO annotation based on both computational predictions and extended manual curation has so far only been available for one of its species, namely *A. nidulans*.

**Results:**

Based on protein homology, we mapped 97% of the 3,498 GO annotated *A. nidulans *genes to at least one of seven other *Aspergillus *species: *A. niger*, *A. fumigatus*, *A. flavus*, *A. clavatus*, *A. terreus*, *A. oryzae *and *Neosartorya fischeri*. GO annotation files compatible with diverse publicly available tools have been generated and deposited online. To further improve their accessibility, we developed a web application for GO enrichment analysis named FetGOat and integrated GO annotations for all *Aspergillus *species with public genome sequences. Both the annotation files and the web application FetGOat are accessible via the Broad Institute's website (http://www.broadinstitute.org/fetgoat/index.html). To demonstrate the value of those new resources for functional analysis of omics data for the genus *Aspergillus*, we performed two case studies analyzing microarray data recently published for *A. nidulans*, *A. niger *and *A. oryzae*.

**Conclusions:**

We mapped *A. nidulans *GO annotation to seven other *Aspergilli*. By depositing the newly mapped GO annotation online as well as integrating it into the web tool FetGOat, we provide new, valuable and easily accessible resources for omics data analysis and interpretation for the genus *Aspergillus*. Furthermore, we have given a general example of how a well annotated genome can help improving GO annotation of related species to subsequently facilitate the interpretation of omics data.

## Background

Gene Ontology (GO) is a framework for functional annotation of gene products aiming to provide a unique vocabulary for living systems [1]. It comprises Biological Process (BP), Molecular Function (MF) and Cellular Component (CC) ontologies. GO terms are organized as directed acyclic graphs (DAG) meaning that GO terms are connected as nodes by directed edges defining hierarchical parent-child relationships. As a consequence, the specificity of GO terms increases with increasing distance from their root node. Enrichment analysis of GO terms is a well accepted approach to dissecting omics data in a non-biased manner. It has been used in many studies to highlight major trends in genomic, transcriptomic or proteomic datasets and describe them with a controlled vocabulary [2-5]. If the frequency of specific GO terms in a list of genes or proteins is higher than expected by chance, it is likely that these enriched GO terms are related to the biological processes under investigation.

The genus *Aspergillus *covers a group of filamentous fungi that includes saprophytes, human and plant pathogens as well as species being exploited in biotechnology. Whereas *A. nidulans *has been comprehensively studied and used as model organism, *A. niger*, *A. oryzae *and *A. terreus *are important industrial workhorses for the production of various enzymes and organic acids. In medical research, *A. fumigatus *and *Neosartorya fischeri *are intensively studied because of their importance as allergens and pathogens of immunocompromised patients. The aflatoxin producing fungus *A. flavus *is well known to cause spoilage of a great variety of agricultural goods. With genome sequences publicly available for eight of its species, the genus *Aspergillus *provides an important group of related fungal species for comparative genomics [6]. The exceptional role of this genus in the genomics of filamentous fungi is further emphasized by a community sequencing project (CSP#350), which has recently been initiated by the DOE Joint Genome Institute (JGI), aiming to sequence nine additional *Aspergillus *species. However, despite the importance of the genus *Aspergillus, A. nidulans *has so far been the only species with a genome-scale GO annotation inferred from both orthology mapping and intense manual curation [7-9], thus providing a valuable resource for the analysis of omics data.

In this work, we have generated a new central repository for functional analysis of omics data for the genus *Aspergillus *using GO annotation. Firstly, we extended the GO annotation of *A. nidulans *to all *Aspergillus *species with publicly available genome sequences and generated annotation files compatible with diverse publicly available tools for GO enrichment analysis. Secondly, we further improved the accessibility of the GO annotation for the genus *Aspergillus *by integrating it into a web tool for GO enrichment analysis and graph visualization named Fisher's exact test Gene Ontology annotation tool (FetGOat). Finally, we performed two case studies to demonstrate the value and flexibility of the newly generated resources for functional analysis of omics data for the genus *Aspergillus*.

## Results

### Mapping of GO annotation

*A. nidulans *is the only *Aspergillus *species for which comprehensive GO annotation based on both computational prediction and extended manual curation of gene-specific literature is available [9]. It constitutes a valuable resource for GO enrichment analysis, which has proven to be a powerful tool for dissecting omics data, for example sets of differentially expressed genes. The GO annotation of *A. nidulans *available at the Aspergillus Genome Database (AspGD) [9] covers 33% (3,498) of its predicted transcripts and associates them with 3,340 GO terms. Including all parental nodes, the list of GO terms extends to 5,508 comprising 3,061 (55%) BP, 1,753 MF (32%) and 694 (13%) CC terms.

**Figure 1:**
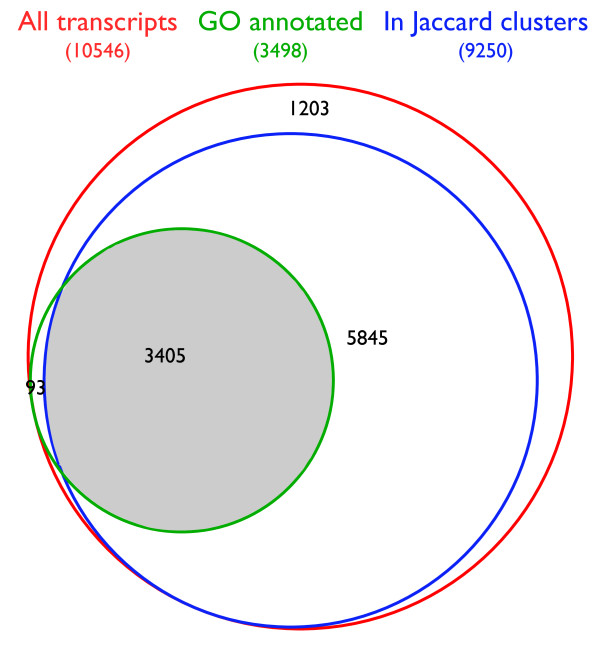
**Mapping *A. nidulans *GO annotation to Jaccard orthologous clusters**. Area-proportional Venn diagram [39] showing fractions of all *A. nidulans *transcripts (red) annotated by GO (green) and/or associated with Jaccard orthologous protein clusters (blue). The intersection of all circles (gray), comprising 3405 transcripts, was used to map *A. nidulans *GO annotation to seven other *Aspergillus *species.

To extend this valuable resource to other species of its genus, we mapped the *A. nidulans *GO annotation to all *Aspergillus *strains with published genome sequences (see Table 1). Groups of orthologous and close paralogous proteins were compiled with the Sybil comparative analysis package [10], which applies a modified reciprocal best-hit approach comprising two clustering cycles. Roughly 89% (99,679) of all predicted proteins from the ten analyzed *Aspergillus *strains constituted 13,179 Jaccard orthologous clusters. For *A. nidulans*, 9,250 of its predicted proteins were organized in Jaccard orthologous clusters, meaning that roughly 80% of all *A. nidulans *proteins were linked to at least one ortholog of another *Aspergillus *species. Of the 3,498 GO annotated *A. nidulans *genes, 97% were contained in Jaccard orthologous clusters, meaning that their associated annotations could be mapped to at least one other *Aspergillus *species (see Figure 1). Overall, mapping resulted in an average of 3,484 GO annotated transcripts per genome ranging from 3,403 (*A. clavatus*) to 3,574 (*A. flavus*). On average, their GO annotations comprise 5,436 terms, (see Table 1). These numbers correspond well to the GO annotation of *A. nidulans *and indicate that the majority (97%) of the *A. nidulans *GO annotated genes could be efficiently mapped to the other *Aspergilli*.

**Table 1:** Mapping of *A. nidulans *GO annotation

		Transcripts		
			
Species	Strain	Predicted	GO annotated (%)	GO terms
*A. nidulans*	FGSC A4	10546	3498 (33)	5508
*A. fumigatus*	AF2937	9846	3443 (35)	5445
*A. fumigatus*	A1163	10109	3450 (34)	5446
*A. flavus*	NRRL 3357	13487	3574 (26)	5463
*A. niger*	CBS 513.88	14366	3540 (25)	5430
*A. niger*	ATCC 1015	11200	3487 (31)	5412
*A. oryzae*	RIB40	12319	3502 (28)	5434
*A. terreus *	NIH 2624	10402	3414 (33)	5406
*A. clavatus*	NRRL 1	9379	3403 (36)	5449
*N. fischeri*	NRRL 181	10728	3543 (33)	5445

Summary of mapping *A. nidulans *GO annotation to seven other *Aspergilli*. The number of predicted transcripts were obtained from the Central Aspergillus Data REpository (CADRE) [40].

### Availability of GO resources for the genus *Aspergillus*

The newly mapped GO annotations were deposited at the Broad Institute's website (http://www.broadinstitute.org/fetgoat/index.html). Different annotation file formats were generated that can be used with diverse public tools for GO enrichment analysis, such as: the Gene Set Enrichment Analysis tool (GSEA) [11], the functional annotation suite Blast2GO [12], the Cytoscape plug-in BiNGO [13] and the Bioconductor package TopGO [14]. To further improve its accessibility, we have implemented Fisher's exact test [15], a well-accepted approach for GO enrichment analysis, in the web application FetGOat and integrated the newly mapped GO annotations. FetGOat can be accessed via a web interface at the Broad Institute's website (http://www.broadinstitute.org/fetgoat/index.html). It combines GO annotations for all *Aspergillus *species with public genome sequences and a widely used statistical methodology to identify overrepresented GO terms. Via the web interface, a list of gene identifiers can be uploaded to the server and statistical parameters can easily be adjusted with end-user computational skills. After completion of the analysis on the server-side, the enrichment results are sent by Email. The results consist of plain text and spreadsheet files as well as scalable vector graphics representing graphs of enriched GO terms.

### Case studies

To demonstrate the flexibility and value of the newly generated resources for omics data analysis, we performed two case studies analyzing transcriptomic datasets recently published for the genus *Aspergillus*. In the first case study, we demonstrate that the generated resources can be used with various methods for enrichment analysis. We analyze a set of maltose-induced genes from *A. niger *using FetGOat and two alternative tools for enrichment analysis to subsequently compare their results. In the second case study, we highlight the advantage of having GO annotations that are as comprehensive as possible available for different species. We use FetGOat to analyze sets of glycerol-induced genes derived from a three-species microarray study to highlight major differences in the transcriptional responses for *A. nidulans*, *A. niger *and *A. oryzae*.

#### Maltose-induced genes

The first dataset reflects the transcriptomic responses of *A. niger *to growth in maltose and xylose-limited chemostat cultures at identical growth rates. From manual analysis of roughly 700 upregulated genes, Jørgensen *et al*. [16] concluded a concerted induction of secretory pathway genes in maltose compared to xylose-limited cultures.

Using three alternative approaches, we repeated the analysis of the maltose induced genes in an automated and un-biased manner to subsequently compare their enrichment results. First, we performed the analysis using the web application FetGOat. We identified 73 enriched GO terms, which were reduced to 19 most-specific GO terms by removing redundant higher hierarchy terms with less detailed annotations. In correspondence to the findings by Jørgensen *et al*., the enriched GO terms are related to important steps involved in protein secretion: Translocation to the endoplasmic reticulum, glycosylation and transport between the endoplasmic reticulum and the Golgi apparatus (see Table 2).

**Table 2:** FetGOat enrichment analysis of maltose-induced genes

				Transcripts	
				
GO term	Description	FDR	Ontology	Induced	Predicted
*Translocation to ER*					
GO:0031204	posttranslational protein targeting	2.7E-04	BP	6	6
GO:0031207	Sec62/Sec63 complex	6.1E-03	CC	3	3
GO:0005787	signal peptidase complex	6.1E-03	CC	3	3
GO:0006616	SRP-dependent cotranslational protein targeting	1.7E-02	BP	4	5
GO:0051605	protein maturation by peptide bond cleavage	1.6E-02	BP	5	8
*Glycosylation in ER*					
GO:0005788	endoplasmic reticulum lumen	3.3E-03	CC	4	5
GO:0008250	oligosaccharyltransferase complex	6.9E-03	CC	4	6
GO:0006487	protein amino acid N-linked glycosylation	4.2E-02	BP	8	24
GO:0016758	transferase activity, transferring hexosyl groups	3.4E-02	MF	14	54
*Transport between ER and golgi*					
GO:0030126	COPI vesicle coat	1.3E-02	CC	4	7
GO:0030127	COPII vesicle coat	6.9E-03	CC	4	6
GO:0006888	ER to Golgi vesicle-mediated transport	4.8E-03	BP	22	92
GO:0030173	integral to Golgi membrane	12.0E-02	CC	5	12
*Starch metabolism*					
GO:0005982	starch metabolic process	4.2E-02	BP	5	10
*Miscellaneous*					
GO:0006066	alcohol metabolic process	4.2E-02	BP	33	199
GO:0003756	protein disulfide isomerase activity	8.2E-03	MF	4	4
GO:0006083	acetate metabolic process	4.2E-02	BP	7	19
GO:0015812	gamma-aminobutyric acid transport	4.2E-02	BP	6	14
GO:0015935	small ribosomal subunit	5.6E-03	CC	12	44

Most specific GO terms identified by FetGOat as being enriched in the maltose-induced gene set. GO terms were grouped in five arbitrary categories: Translocation into endoplasmic reticulum (ER), glycosylation in ER, transport between ER and golgi, starch metabolism and miscellaneous.

For comparison of FetGOat with alternative programs, we used the generated annotation files and repeated the enrichment analysis with two publicly available tools, Blast2GO [12] and GSEA [11]. The numbers of enriched GO terms found with Blast2GO and GSEA are in the same range compared to the results from FetGOat, they identified 76 and 47 enriched GO terms, respectively. To compare the enrichment results from the three tools, we computed semantic similarity scores with the G-SESAME tool [17]. For both FetGOat and Blast2GO, the enrichment statistic is based on Fisher's exact test and thus their results are theoretically expected to be identical resulting in a semantic similarity score of 1. A similarity score of 0.983 confirms that their results are virtually identical, with minor differences that are likely due to differences in their implementations. In contrast to FetGOat (and Blast2GO), the GSEA results are based on running-sum statistics computed from the complete expression data set. Therefore, the similarity between their results can be expected to be less. Accordingly, G-SESAME determined a smaller semantic similarity score of 0.863 for the results obtained with FetGOat and the GSEA tool.

In addition to the GO terms identified by both Fisher's exact test based tools, GSEA computed an enrichment of GO terms related to oxidative phosphorylation (GO:0006119), carbohydrate transport (GO:0008643) and glucosidase activity (GO:0015926). Comparing maltose to xylose limitation, an enrichment of those GO terms fits our expectations. Under maltose-limitation, *A. niger *breaks down the disaccharide into its monomer glucose by enzymes having glucosidase activity. Subsequently, glucose is taken up by carbohydrate transporters, which can be expected to be different from those required for the uptake of xylose. Finally, 1 mole of glucose yields more ATP than 1 mole of xylose, thereby explaining an induction of oxidative phosphorylation.

These differences in the enrichment results are potentially inherited by the statistics applied by Jørgensen *et al*. to define the set of maltose-induced genes. In contrast to the GSEA tool, which analyzes the complete expression data, FetGOat and Blast2GO are depending on a-priori performed statistics that were applied to generate subsets of genes or proteins of interest. Jørgensen *et al*. used the Affymetrix MAS 5.0 algorithm for data pre-processing in combination with the student's t-test to define their set of maltose induced genes. In current literature, this approach is critically discussed [18,19]. To assess the effect of those a-priori applied statistics on the differences between the results from FetGOat and the GSEA tool, we generated an alternative set of maltose-induced genes. We computed RMA expression data [18] from the raw data (CEL files) and subsequently applied a moderated t-statistic [20] to identify upregulated genes (data not shown). Interestingly, FetGOat also identified enriched GO terms related to glucosidase activity and carbohydrate transport for this alternative set of maltose-induced genes. However, no enrichment of genes related to oxidative phosphorylation was found. Genes annotated with the GO term oxidative phosphorylation were only marginally induced and their FDR values were rather high (data not shown). Interestingly, similar differences between Fisher's exact test based methods and the GSEA tool were reported in another study. In muscle tissue from diabetics, the GSEA tool identified a joint downregulation of genes related to oxidative phosphorylation compared to healthy controls, while no enrichment was found in the set of downregulated genes [21]. For tightly regulated essential cellular processes that show only minor fold changes, the GSEA tool seems to be superior to gene-by-gene differential expression studies.

#### Glycerol-induced genes

In the second case study, we used FetGOat to analyze transcriptomic data generated by Salazar *et al*. [22]. With a three-species microarray, the authors studied the transcriptomic responses of *A. nidulans*, *A. niger *and *A. oryzae *to growth in glycerol and glucose-limited batch cultures. The authors identified 4,139 glycerol-induced genes comprising 679, 2,240 and 1,040 genes from *A. nidulans*, *A. niger *and *A. oryzae*, respectively. Based on tri-directional best blast hits, 81 orthologous gene clusters were shown to be upregulated in each of the species. Using the *A. niger *(strain ATCC 1015) GO annotation, Salazar *et al*. analyzed the set of conserved upregulated genes and identified enriched BP terms, which are related to amino acid metabolism, gluconeogenesis, hexose and alcohol biosynthetic processes.

**Figure 2:**
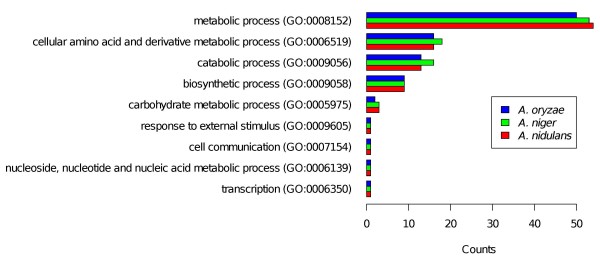
**Comparative GO enrichment analysis of conserved glycerol-induced orthologous gene sets**. Comparative enrichment analysis of 81 glycerol-induced and conserved genes of the three species *A. nidulans*, *A. niger *and *A. oryzae *[22]. Using FetGOat, enrichment analysis of BP terms was performed independently for each of the orthologous gene sets. For comparison, the enriched GO terms were mapped to a GO Slim annotation and the occurrences of the corresponding GO terms were counted with the CateGOrizer tool [38].

First, we repeated the enrichment analysis similar to Salazar *et al*. on the set of 81 upregulated and conserved genes. With the web application FetGOat, we individually performed enrichment analysis using GO annotations of *A. nidulans*, *A. niger *(strain ATCC 1015) and *A. oryzae*. FetGOat identified 58, 57 and 54 enriched BP terms, respectively. To summarize the enrichment results for the three *Aspergilli *and compare them with each other, we mapped the GO terms to a GO Slim annotation and counted the occurrences of related GO terms. As expected from analyzing orthologous gene sets, the counts for the GO Slim terms were nearly identical, independent of which of the three *Aspergilli *the enrichment analysis was performed for (see Figure 2). To further assess the similarity of the three lists of enriched GO terms, we used the G-SESAME tool [17] and computed pair-wise semantic similarity scores for *A. nidulans *vs. *A. niger*, *A. nidulans *vs. *A. oryzae *and *A. niger *vs. *A. oryzae *of 0.991, 0.992 and 0.993, respectively. The similarity of the three enrichment results indicates that the newly mapped GO annotations for *A. niger *and *A. oryzae *are well comparable with each other and the *A. nidulans *GO annotation.

Corresponding to the enrichment results from Salazar *et al*., FetGOat identified enriched GO terms that are related to pyruvate and (aromatic) amino acid metabolism. Unlike Salazar *et al*., FetGOat did not identify BP terms related to gluconeogenesis. This difference can be explained by an improvement of the GO annotation. While only three genes were annotated with the BP term gluconeogenesis (GO:0006094) in the GO annotation used by Salazar *et al*., it is a total of 28 genes in the newly mapped GO annotation for *A. niger *(ATCC 1015 strain). For both annotations, one out of the upregulated conserved genes is annotated by the BP term gluconeogenesis, thus explaining why Salazar *et al*. identified it as an enriched BP term and FetGOat did not.

**Figure 3:**
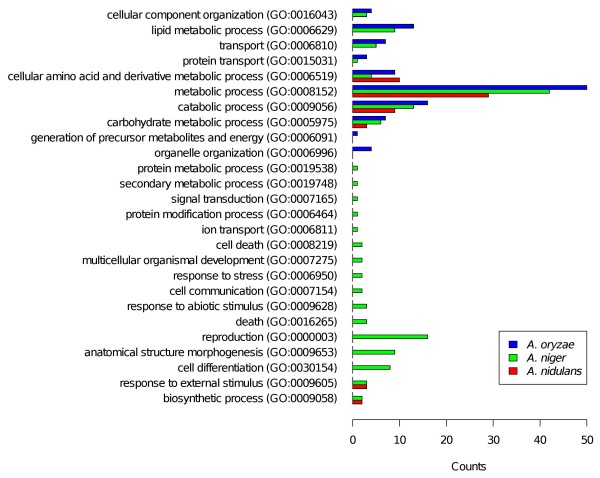
**Comparative GO enrichment analysis of individual glycerol-induced gene sets**. FetGOat was used for comparative enrichment analysis of the complete glycerol-induced gene sets of the three species *A. nidulans*, *A. niger *and *A. oryzae *[22]. For comparison, the enriched GO terms were mapped to a GO Slim annotation and the occurrences of the corresponding GO terms were counted with the CateGOrizer tool [38].

Next, we aimed to identify differences in the tendencies of the transcriptional responses to glycerol for the three *Aspergilli*. With FetGOat, we individually performed enrichment analysis on each of the complete sets of upregulated genes and found 35, 100 and 65 enriched BP terms for *A. nidulans*, *A. niger *and *A. oryzae*, respectively. The differences in the number of enriched BP terms correspond to the differences in the number of upregulated genes. To summarize and compare the results with each other, we mapped the GO terms to a GO-Slim annotation and counted their occurrences (see Figure 3). This summary clearly shows different tendencies in the transcriptomic responses of the three *Aspergilli*. Most strikingly, a number of GO-Slim terms were identified as being enriched for *A. niger *but not for the other two *Aspergilli*. Many of the associated GO terms are directly or indirectly related to nutrient limitation such as conidiation, secondary metabolic processes and cell death. Furthermore, FetGOat found an enrichment of the BP term response to nutrient levels (GO:0031667) for *A. nidulans *(nine upregulated genes) and *A. niger *(30 upregulated genes) but not *A. oryzae*. In contrast, GO terms related to energy generation and peroxisomal organization were enriched for *A. oryzae *but not for the other two *Aspergilli*. FetGOat further computed an enrichment of the BP term carbohydrate transport (GO:0008643) specifically for *A. oryzae*. Interestingly, the different transcriptional trends correspond well with the physiological data. The capacities to grow on glycerol differ significantly for the three *Aspergilli*. With a maximum specific growth rate of 0.05 *h*^-1^, which is one-fourth of its maximum specific growth rate on glucose, *A. niger *grew the worst on glycerol. In contrast, *A. oryzae *showed the fastest growth (0.30 *h*^-1^), which is equal to approximately 80% of its maximum specific growth rate on glucose. *A. nidulans *is in between and grew with roughly 50% of its glucose specific speed.

## Discussion

A detailed and comprehensive genome annotation can be considered a prerequisite for the analysis and interpretation of omics data. GO provides a framework for functional annotation and has been proven to be a valuable tool for omics data analysis, especially in combination with enrichment statistics. Currently, the GO reference genome project [23] provides the most comprehensive manually curated GO annotation for twelve model organisms and is intended to serve as a reference for automated mapping of GO annotation to organisms other than these major models. From the reference genome projects, *Saccharomyces cerevisiae *and *Schizosaccharomyces pombe *are most closely related to the genus *Aspergillus*.

*A. nidulans *has so far been the only *Aspergillus *species with comprehensive genome scale GO annotation based on both orthology mapping to *S. cerevisiae *and extensive manual curation [9] of gene-specific literature. We have thus mapped the *A. nidulans *GO annotation to all other *Aspergillus *species (see Table 1) with published genomes. With 79% of all *A. nidulans *genes being organized in Jaccard orthologous clusters covering 97% of all its GO annotated genes, we demonstrated that this approach is promising for mapping GO annotation between closely related genomes such as those of the genus *Aspergillus*. Nevertheless, the newly generated GO annotations have exclusively been inferred by computational analysis and thus their quality can be expected to be lower compared to the extensively manually curated *A. nidulans *GO annotation. The ortholog clustering approach as implemented in the Sybil comparative analysis package [10] has worked well for a number of comparative genome studies [24-33], but does have limitations, especially when there are a large number of strains and/or percentage of repetitive proteins. Additionally, we recognize that the optimal choice of an ortholog detection method depends on the purpose of the analysis. This graph based approach is robust if looking at closely related species, but may not be the best choice when considering large numbers of more distantly related genomes.

The GO annotations for ten *Aspergillus *strains (see Table 1) have been made available at the Broad Institute's website (http://www.broadinstitute.org/fetgoat/index.html) and will be updated regularly as the GO annotations for the various *Aspergillus *species continue to improve through manual and computational efforts. To improve the applicability of the GO annotations, they are provided in different file formats that can be used with various freely available GO enrichment tools, e.g. Blast2GO [12], TopGO [14], GSEA [11] and BinGO [13]. Thereby, functional analysis of *Aspergillus *omics data by GO enrichment analysis is strongly facilitated. The availability of different annotation file formats makes it feasible to use different tools and compare them with each other.

To further improve the accessibility of the extended annotations, we developed the web application FetGOat and integrated the GO annotation for all *Aspergillus *species with public genome sequences. FetGOat basically resembles the functionality of other publicly available enrichment tools. However, for the *Aspergillus *research community, FetGOat is a valuable addition to existing programs because it uniquely combines an intuitive web interface, GO annotations for all *Aspergilli *with public genome sequences and a frequently applied statistical method for the identification of enriched GO terms.

To demonstrate the use of those newly generated resources for functional analysis of omics data, we applied them in two case studies to re-analyze recently published microarray data in an automated and un-biased manner. As shown for the first dataset, the enrichment results are in correspondence to the main conclusions from Jørgensen *et al*. [16]. We found an induction of processes related to secretion, glycosylation and starch degradation (see Table 2). In addition, we used the dataset from Jørgensen *et al*. to compare the enrichment results of FetGOat to those obtained with two well established publicly available tools, Blast2GO and GSEA. The three tools apply two different methods for enrichment analysis. While Blast2GO and FetGOat compute a Fisher's exact test statistic to identify GO terms that are over-represented in subsets of genes derived e.g. from transcriptomic or proteomic data, the GSEA tool computes running sum statistics on (non-filtered) expression data to identify a-priori defined groups of genes that show joined differential expression. The results from FetGOat are virtually identical to the results obtained with Blast2GO demonstrating the correctness of FetGOat. As expected, the similarity between the results from FetGOat and the GSEA tool is less, while their results are still well comparable. For a large part, both tools are highlighting the same transcriptional trends. However, the GO term oxidative phosphorylation was exclusively identified as being enriched by the GSEA tool. Taking into account that 1 mole of glucose yields more ATP than 1 mole of xylose, an induction of the oxidative phosphorylation machinery during growth in maltose-limited cultures can be expected. Because the fold-changes of the corresponding genes were very small and their statistical significances were low, no enrichment could be found in the set of maltose-induced genes as assessed by Fisher's exact test. Similar results were found in another study, in which the GSEA tool detected a joined transcriptional downregulation of genes related to oxidative phosphorylation in tissue from diabetics vs. control [21]. For tightly regulated essential genes, which show only marginal differential expression, the GSEA tool seems to be superior to gene-by-gene differential expression approaches. However, we would like to emphasize that this is rather caused by the a-priori performed statistics than by the Fisher's exact test itself. A combination of clustering based on gene expression profiles combined with Fisher's exact test enrichment statistics will potentially allow to draw similar conclusions as with the GSEA tool. The causality between an increased ATP yield for maltose and an upregulation of secretion related genes remains to be investigated. However, it is an interesting new hypothesis for further investigations.

For the second dataset from Salazar *et al*. [22], we first performed GO enrichment analysis on the set of 81 conserved and glycerol-induced genes used in the original study. We could partly reproduce the enrichment results. However, we didn't find an enrichment of genes annotated with the GO term gluconeogenesis. A comparison of the GO annotation used by Salazar *et al*. and our newly mapped GO annotation revealed that this is due to an improvement of the newly mapped GO annotation, which includes many more genes annotated with the GO term gluconeogenesis. As expected from analyzing orthologous gene sets, we showed that the enrichment results are nearly identical, independent of which of the three *Aspergilli *they were obtained for. Furthermore, we separately performed enrichment analysis for the three *Aspergilli *analyzing their complete sets of up regulated genes and highlighted major differences in their responses to glycerol vs. glucose limitation. Thereby, we were able to draw additional conclusions explaining their different capabilities to grow on glycerol. Especially for the three-species microarray platform, FetGOat in combination with the newly mapped GO annotation forms a new, valuable and flexible resource for omics data analysis. Applied at an early stage of data analysis, GO enrichment analysis can thus strongly facilitate subsequent manual data interpretation.

While GSEA is an attractive alternative to Fisher's exact test based tools such as FetGOat and Blast2GO, it lacks flexibility because it is restricted to transcriptomic data and can only compare two conditions at a time. Furthermore, its application is more sophisticated, because microarray specific chip annotation files as well as phenotypic labels have to be provided for analysis. Tools such as FetGOat and Blast2GO can be applied to any set of genes or proteins deriving from genomic, transcriptomic or proteomic studies. They can for example be used to perform GO enrichment analysis on a set of proteins commonly secreted under certain conditions. Improving the power of the statistics applied to obtain gene sets of interest will consequently improve the strength of Fisher's exact test based enrichment analysis. For transcriptomic data analysis, moderated statistics or non specific filtering have for example been shown to improve the statistical power [19].

The choice of a tool for GO enrichment analysis depends on the type of data, the available resources and personal preferences. Certainly, most of the enrichment results will be redundant between the tools. With the different GO annotation files generated in this study, various freely available tools can easily be used and compared with each other. Especially for the genus *Aspergillus*, FetGOat stands out with respect to the ease of use and the integration of comprehensive and regularly updated GO annotations. The power of FetGOat lies in its flexibility. Any set of genes/proteins from any *Aspergillus *strain with published genome sequence can be investigated for enrichment of GO terms. FetGOat is not restricted to the genus *Aspergillus *as it can be extended to include GO annotations from any organism of interest.

## Conclusions

We have mapped the *A. nidulans *GO annotation to the genomes of seven other *Aspergillus *species and made the GO annotations available in different file formats. We furthermore developed the web tool FetGOat, which can be used for GO enrichment analysis of omics data from all *Aspergillus *strains with published genome sequences. Both, the mapped GO annotations and FetGOat were successfully applied in two case studies and are available at the Broad Institute's website (http://www.broadinstitute.org/fetgoat/index.html). Moreover, we have given a general example of how a well annotated genome can help improving GO annotation of related species to subsequently facilitate the interpretation of omics data.

## Methods

### Ortholog and paralog identification

Clusters of orthologous proteins from ten *Aspergillus *strains (see Table 1) were generated with Sybil [10]. The Sybil comparative analysis package currently utilizes the following two-step clustering method, which is a modification of the standard reciprocal best match approach. First, an all-vs-all protein similarity matrix is computed by searching each of the predicted polypeptides within the genomes being compared against all polypeptides. BLASTP is currently used for these searches, with an E-value cut off of 1E-5. Polypeptides from each individual species are clustered independently using only BLASTP hits that had a sequence identity score of at least 80%. The BLASTP matches that meet these criteria are used to compute a Jaccard similarity coefficient [34] for each distinct pair of polypeptides in the same genome. Given two polypeptides P1 and P2 the Jaccard similarity coefficient is defined as:

$$J\left(P1,P2\right)=\frac{\text{matches to}\;P1\cap P2}{\text{matches to}\;P1\cup P2}$$

Using default parameters, any pair of polypeptides with a Jaccard coefficient > 0.6 is connected in a graph representation. The connected components of this graph are referred to as "Jaccard Clusters" and are analogous to paralogous protein clusters within each species. Subsequently, the reciprocal best-hit phase of the clustering algorithm identifies pairs of Jaccard clusters such that: (1) The clusters are from different genomes. (2) The highest-scoring BLASTP match of at least one polypeptide in each of the clusters is to a polypeptide in the other cluster. A graph is constructed, with an edge drawn between two nodes (Jaccard clusters) if and only if they are bidirectional best BLASTP matches of each other. The connected components of this graph are considered ortholog groups in downstream analysis and will be referred to as "Jaccard orthologous clusters".

### Mapping *A. nidulans *GO annotation

GO annotation for *A. nidulans *(gene_association.aspgd version: 1.256) was obtained from the Aspergillus Genome Database (AspGD: http://www.aspgd.org) [9] and is based on orthology mapping between *A. nidulans *and *S. cerevisiae *as well as extensive manual curation based on gene specific *A. nidulans *literature. GO terms for Jaccard orthologous clusters and their associated proteins were inferred from *A. nidulans *GO annotation such that each protein belonging to the same Jaccard orthologous cluster shares identical GO terms. For each of the analyzed strains (see Table 1), individual GO annotation files were generated in different formats.

### Enrichment analysis

GO enrichment analyses were performed applying two different statistical tests: Fisher's exact test [15] and Kolmogorow-Smirnov statistics [11,35]. If not stated differently, p-values were corrected according to Benjamini & Hochberg [36] and a critical False Discovery Rate (FDR) q-value of 0.05 was applied. For the Fisher's exact test based enrichment analysis of GO terms, we developed the web application FetGOat, which calculates one-tailed p-values and corrects them for multiple hypothesis testing according to the Benjamini & Hochberg method. In addition to FetGOat, Blast2GO [12] was used to compute enriched GO terms via Fisher's exact test as implemented in GOSSIP [37]. For the identification of enriched GO terms based on the Kolmogorov-Smirnov statistic, the GSEA tool [11] was used. The corresponding GO annotation files for Blast2GO and the GSEA tool were generated in this study and are available at the Broad Institute's website (http://www.broadinstitute.org/fetgoat/index.html).

### Mapping to GO slim annotation

To summarize GO enrichment results, we mapped the enriched GO terms to a GO Slim annotation [1], which is a reduced version of the complete annotation with less detailed high-level GO terms, and counted the occurrences (single occurrence option) of GO Slim terms as well as related lower hierarchy terms using the CateGOrizer tool [38].

## Authors' contributions

BMN implemented a data analysis pipeline to map GO annotation to Jaccard clusters and generate annotation files, developed FetGOat and performed further data analysis. JC and JRW generated Jaccard clusters. GCC integrated FetGOat at the Borad Institute's website. JRW, AFJR and VM were involved in writing the manuscript. All authors read and approved the final manuscript.
